# ﻿Description of two new species of *Cyrtodactylus* Gray, 1827 (Squamata, Gekkonidae) from Nepal

**DOI:** 10.3897/zookeys.1253.161933

**Published:** 2025-09-23

**Authors:** Santosh Bhattarai, Bivek Gautam, Bishal Prasad Neupane, Akshay Khandekar, Tejas Thackeray, Ishan Agarwal, Ashley R. Olson, Fiona Hogan, Wendy Wright

**Affiliations:** 1 Future Regions Research Centre, Federation University Australia, Gippsland Campus, Churchill 3842, Victoria, Australia. Nepal Conservation and Research Center Chitwan Nepal; 2 Nepal Conservation and Research Center, Ratnanagar-06, Chitwan, Nepal Federation University Australia Churchill Australia; 3 Biodiversity Research and Conservation Society, Kathmandu, Nepal Biodiversity Research and Conservation Society Kathmandu Nepal; 4 Department of Zoology, Shivaji University, Kolhapur, 41600, India Shivaji University Kolhapur India; 5 Thackeray Wildlife Foundation, Mumbai, 400051, India Thackeray Wildlife Foundation Mumbai India

**Keywords:** Bent-toed gecko, Himalayas, integrative taxonomy, *khasiensis* group, Siwalik

## Abstract

Two new species of *Cyrtodactylus* from the *khasiensis* group are described using morphological characters supported by molecular analyses based on the mitochondrial ND2 gene. *Cyrtodactylus makwanpurgadhiensis***sp. nov.** and *C. chure***sp. nov.** from the Siwalik Mountains in central Nepal are at least 11.2% divergent from other Nepalese congeners and 16.7% from each other and can be distinguished by a combination of morphometric and meristic traits. The description of two new species from the Siwalik Mountains underscores the conservation significance of this region, a relatively young, dry, and geologically unstable range of the Himalayan orogen. Despite serving as a border between the lowland (Terai) and Himalayan range, it remains underrepresented in both biodiversity assessments and conservation planning in Nepal. Our findings suggest the need for a robust and targeted species research program and to prioritise this landscape for conservation actions.

## ﻿Introduction

**Figure 1.**
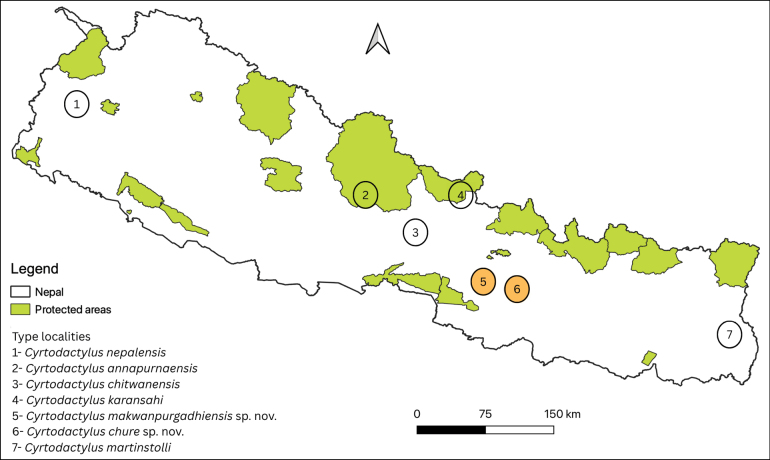
Outline map of Nepal showing type localities of *Cyrtodactylus* spp. and protected area coverage; new species are in orange circles.

Nepal is climatically and topographically diverse with many large mountainous areas that remain unexplored, leaving the country’s biodiversity inventory incomplete (Joshi and Joshi 2022; Bhattarai et al. 2025). A recent review of *Cyrtodactylus* Gray, 1827 (bent-toed geckos) in Nepal, based on recently collected specimens and topotypical material for all described species, included the description of three new species from central Nepal and the synonymy of an existing species (Bhattarai et al. 2025). The review by Bhattarai et al. (2025) placed the five valid *Cyrtodactylus* species from Nepal into two groups: the *fasciolatus* and *khasiensis* (Grismer et al. 2021) within the Indo-Burma clade (Agarwal et al. 2014) — the *fasciolatus* group including the Nepalese *C. chitwanensis* Bhattarai, Gautam, Neupane, Khandekar, Thackeray, Agarwal, Tillack, Olson, Hogan & Wright, 2025 and *C. nepalensis* (Schleich & Kästle, 2002), and the *khasiensis* group including *C. annapurnaensis* Bhattarai, Gautam, Neupane, Khandekar, Thackeray, Agarwal, Tillack, Olson, Hogan & Wright, 2025, *C. karanshahi* Bhattarai, Gautam, Neupane, Khandekar, Thackeray, Agarwal, Tillack, Olson, Hogan & Wright, 2025, and *C. martinstolli* (Darevsky, Helfenberger, Orlov & Shah, 1997) (Bhattarai et al. 2025). The elevational range known to be occupied by *Cyrtodactylus* in Nepal Himalaya is ~600–2000 m above sea level (a.s.l.) and large tracts of suitable habitats within this range remain poorly surveyed. During June–July 2024, we conducted field surveys and collected specimens from Makwanpur and Sindhuli districts of Bagmati Province in central Nepal (Fig. 1). Mitochondrial sequences of these populations are deeply divergent from known species of the Indo-Burma clade, and we describe two new species of *Cyrtodactylus* below.

## ﻿Materials and methods

### ﻿Field survey and sample collection

Field surveys were conducted in Makwanpur and Sindhuli districts of Bagmati Province in central Nepal during June–July 2024 by SB and team, as part of SB’s PhD fieldwork, with the approval of Federation University’s Animal Ethics Committee (AEC-2022-008). All field surveys were conducted after dark in potential *Cyrtodactylus* habitat such as roadside walls, boulders, vegetation, and stone walls of the ancient forts Makwanpurgadhi and Hariharpurgadhi. *Cyrtodactylus* specimens were hand-collected, photographed while alive and later euthanised. Samples of either liver tissue or tail tips were collected from euthanised specimens and stored in 100% ethanol until they could be transported for subsequent long-term storage at -20 °C. Whole specimens were fixed in 8–14% formalin for ~12–24 hours and later transferred to 70% ethanol after being thoroughly washed. Collection permits for this work were issued by the Nepal Government Department of National Parks and Wildlife Conservation and Department of Forests and Soil Conservation (see acknowledgements). Specimens are deposited in the Natural History Museum, Kathmandu, Nepal (**NHM**).

### ﻿Molecular data and analysis

We extracted DNA from thawed tissue using Qiagen® DNeasy Blood and Tissue Kit, with primers L4437 + H5540 (Macey et al. 1997) used to target the mitochondrial protein coding gene ND2 (NADH dehydrogenase 2; 1038 nucleotides). Extractions and Polymerase Chain Reaction (PCR) were carried out at the National Trust for Nature Conservation (NTNC)- Biodiversity Conservation Center, Sauraha, Chitwan District, Bagmati Province, Nepal. Sanger sequencing was conducted by Barcode Biosciences in Bangalore and chromatograms were assembled using Chromas 2.6.6 (Technelisium, Australia; http://technelysium.com.au/wp/chromas/). Newly generated ND2 sequences were deposited in GenBank with accession numbers PX115889–PX115893.

Sequences were aligned with default settings using ClustalW (Thompson et al. 1994) in MEGA 5.2 (Tamura et al. 2011) with translation to amino acid to ensure absence of stop codons. Uncorrected pairwise sequence divergences (p-distance) using the pairwise deletion option were calculated in MEGA. Preliminary analyses placed our new sequences within the Indo-Burma clade, and thus the final alignment included published sequences for the Indo-Burma clade, with the *lawderanus* group (Grismer et al. 2021) used as the outgroup (using the same sequences as Bhattarai et al. 2025: table 1). The best-fitting models of sequence evolution and optimal partition scheme for the data partitioned by codon were estimated using the Bayesian Inference Criterion in PartitionFinder 2 (Lanfear et al. 2016) which selected GTR+I+G for all three codon positions with parameters unlinked across partitions. A Maximum Likelihood (ML) tree was built using in RaXML HPC 8.2.12 (Stamatakis 2014) with 500 thorough bootstraps (BS) and 10 independent runs. Partitioned Bayesian Inference (BI) analyses were conducted in MrBayes 3.2.7 (Ronquist and Huelsenbeck 2003; Ronquist et al. 2012) with four chains each (one cold and three hot) in two parallel runs with 1,000,000 generations sampled every 100 generations, and convergence determined based on a standard deviation of split frequencies (<< 0.01); with a consensus tree reconstructed after removing the first 25% of trees as burn-in.

### ﻿Morphological and meristic data

The morphological dataset comprised of 50 characters and follows Bhattarai et al. (2025) using a total of 43 specimens of *Cyrtodactylus* from Nepal, including *C. annapurnaensis*, *C. chitwanensis*, *C. karanshahi*, *C. martinstolli*, *C. nepalensis* (see Appendix 1) and the two new species. We recorded colour patterns from photographs of live specimens and a single observer (AK) recorded morphological data using a ZEISS Stereo Discovery V8 dissecting microscope on the left side of the body whenever possible, with bilateral scale counts taken on both sides of each specimen. The following measurements were taken with a Mitutoyo digital caliper (to the nearest 0.1 mm) : snout vent length (**SVL**, from tip of the snout to cloacal opening) ; tail length (**TL**, from cloaca to tail tip) ; tail width (**TW**, measured at tail base) ; lower arm length (**LAL**, from elbow to distal end of wrist; measured by flexing elbow at 90° wherever needed) ; crus length (**CL**, from knee to heel; measured by flexing knee at 90° wherever needed) ; axilla to groin length (**AGL**, from posterior margin of forelimb insertion to anterior margin of hindlimb insertion on the body) ; body height (**BH**, maximum height of body measured at midbody) ; body width (**BW**, maximum width of body measured at midbody) ; head length (**HL**, distance from the retroarticular process to the snout tip) ; head width (**HW**, maximum width of head, measured just behind the eyes) ; head height (**HH**, maximum height of head measured at the level of the eye) ; eye diameter (**ED**, greatest horizontal diameter of eye) ; eye to ear distance (**EE**, distance from anterior edge of ear opening to posterior margin of eye) ; eye to snout distance (**ES**, distance between anterior margin of eye and tip of snout) ; eye to nares distance (**EN**, distance between anterior margin of eye and posterior edge of nostril) ; internarial distance (**IN**, distance between nares measured dorsally from their internal margins) ; interorbital distance (**IO**, shortest distance between left and right supraciliary scale rows in front of orbit) ; and ear length (**EL**, maximum length of ear opening).

The following meristic data were recorded for all specimens : number of internasals (**INS**, number of scales behind rostral and between supranasals) ; number of supralabials (**SL**), and infralabials (**IL**), from rostral and mental, respectively, to posterior-most enlarged scale at angle of the jaw ; supralabials at midorbital position (**SL M**), and infralabials at midorbital position (**IL M**), from rostral and mental, respectively, to below the middle of the eye ; paravertebral tubercles (**PVT**, number of enlarged tubercles between limb insertions counted in a straight line immediately left or right of the vertebral column) ; dorsal tubercle rows (**DTR**, number of longitudinal rows of enlarged tubercles around the body counted at midbody) ; mid ventral scale rows (**MVSR**, counted at midbody between the ventrolateral fold) ; ventral scales 1 (**VS1**, counted on midbody ventral between forelimb and hindlimb insertions) ; ventral scales 2 (**VS2**, counted from the mental to anterior border of the cloacal opening) ; distal subdigital lamellae counted from digital inflection at first phalanx to the claw, excluding the large scale on inflection and including the claw sheath on manus : digit 1 (**DLAMF1**), digit 4 (**DLAMF4**), on pes: digit 1 (**DLAMT1**), digit 4 (**DLAMT4**) , and digit 5 (**DLAMT5**) ; basal subdigital lamellae, counted from digital inflection at first phalanx (including the large scale on inflection) to the base of the digits including all scales that are wider than high; on manus: digit 1 (**BLAMF1**), digit 4 (**BLAMF4**), on pes: digit 1 (**BLAMT1**), digit 4 (**BLAMT4**), and digit 5 (**BLAMT5**) ; total lamellae (**TLAMF1**, **TLAMF4**, **TLAMT1**, **TLAMT4**, and **TLAMT5** are sum of respective basal and distal lamellae for all digits) ; precloacal scales (**PCS**, number of enlarged scales excluding the pore-bearing scales on otherwise pore-bearing precloacal row) ; precloacal pores (**PP**, number of pore-bearing precloacal scales) ; post cloacal tubercles (**PCT**, number of post cloacal tubercles on either side of the tail base).

## ﻿Results

### ﻿Phylogenetic relationships

**Figure 2.**
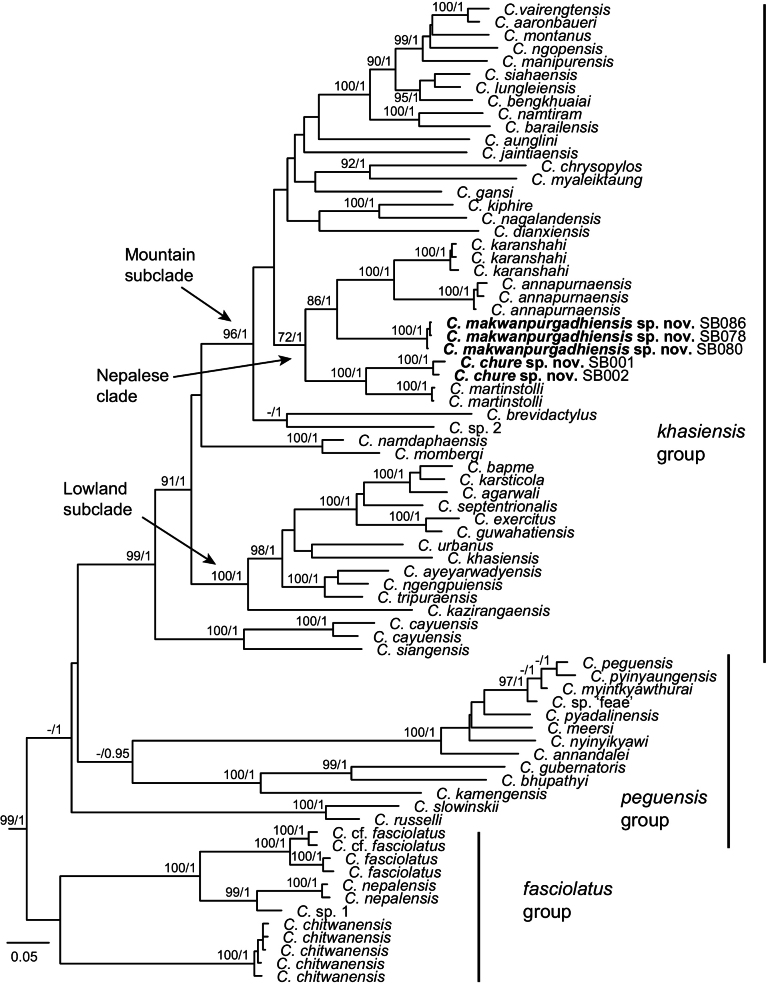
Bayesian phylogram (ND2, 1038 nucleotides) of the Indo-Burma clade of *Cyrtodactylus*; bootstrap support ≥ 70/ posterior probability ≥ 0.95 shown at nodes, specimen numbers for the new species are given, outgroups not shown.

We recover concordant relationships within the Indo-Burma clade as Bhattarai et al. (2025), with the *fasciolatus* group recovered with poor support (BS < 70, posterior probability < 0.95), *khasiensis* group with high support (99/1.00), and a non-monophyletic *peguensis* group (Fig. 2). The additional sequences from the samples collected in central Nepal are related to the previously described Nepalese species within the mountain subclade of the *khasiensis* group, forming a reasonably supported (72/1.00) ‘Nepalese’ clade (Fig. 2). This includes the divergent lineage from Hariharpur forming as the sister taxon to *C. martinstolli*, the two forming a sister group to the clade including the divergent lineage from Makwanpur sister to *C. annapurnaensis* + *C. karanshahi*.

Uncorrected pairwise sequence divergence between the populations from Makwanpur and Sindhuli districts in central Nepal are 16.6–16.9%, 11.7–30.0% compared to previously described Nepalese species, and ≥ 16.3% from previously described species of the Indo-Burma clade (Table 1). We use morphological data to describe and diagnose these two unnamed divergent lineages from central Nepal as new species.

**Table 1.** Uncorrected sequence divergence (%) between Nepalese *Cyrtodactylus* species.

		1	2	3	4	5	6	7
1	*C. makwanpurgadhiensis* sp. nov.	0.5						
2	*C. chure* sp. nov.	16.7	1.9					
3	*C. annapurnaensis*	14.6	19.0	0.0				
4	*C. chitwanensis*	30.0	28.8	30.1	1.7			
5	*C. karanshahi*	16.5	19.0	13.9	29.6	1.4		
6	*C. martinstolli*	15.5	11.7	17.1	29.0	18.6	0.0	
7	*C. nepalensis*	29.3	29.3	28.6	25.9	28.3	28.5	0.5

### ﻿Systematics

#### ﻿ Cyrtodactylus makwanpurgadhiensis sp. nov.

Taxon classificationAnimaliaSquamataGekkonidae

4EA4AE6D-42CC-5E42-A901-1CCBFDA734D2

https://zoobank.org/C4692B76-5A97-4D3E-BFB9-0A18DAD5168F

**Figure 3.**
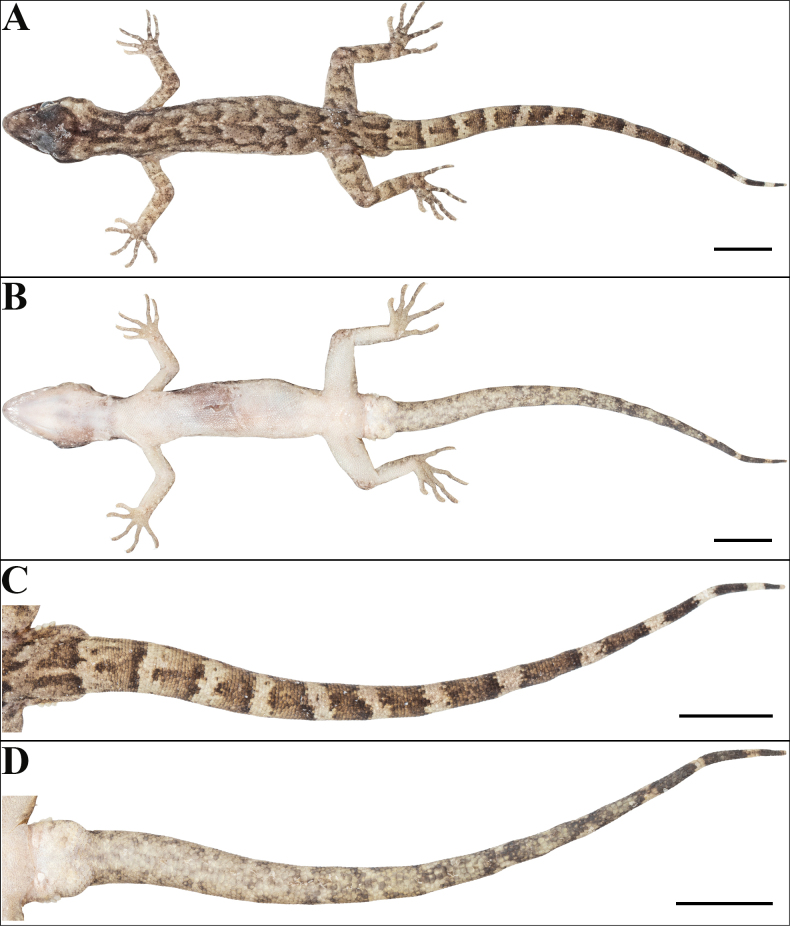
Holotype of *Cyrtodactylus makwanpurgadhiensis* sp. nov. (male, NHM 2025/383): A. Dorsal view of body; B. Ventral view of body; C. Dorsal view of tail, and D. Ventral view of tail. Scale bars 10 mm; photographs by AK.

**Figure 4.**
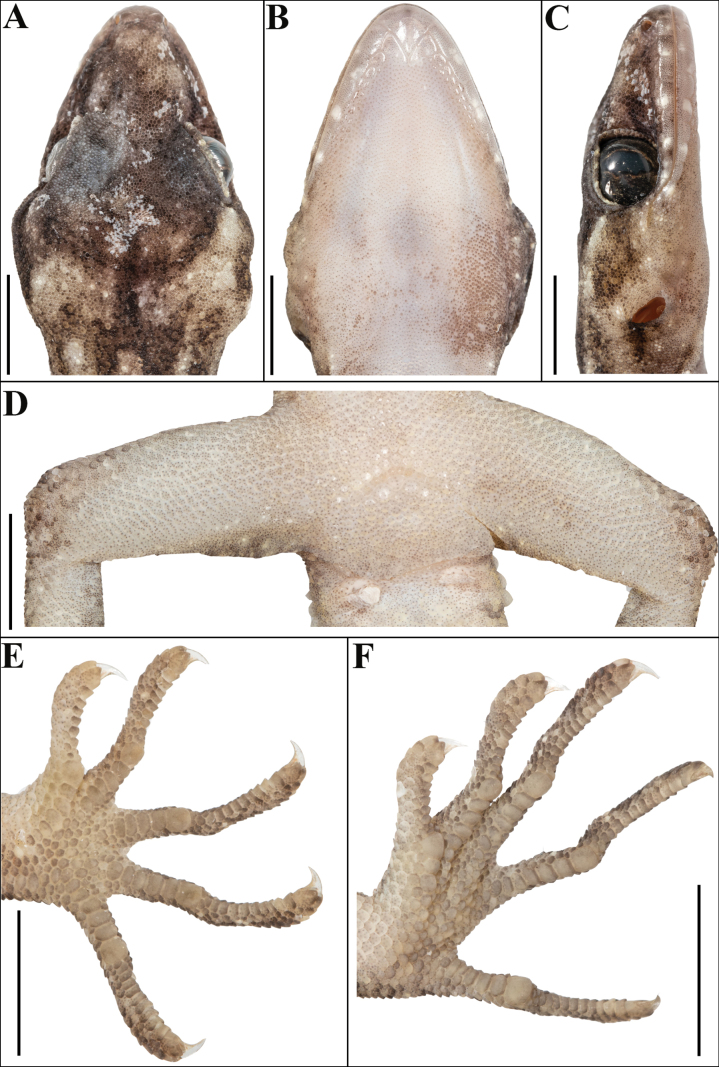
Holotype of *Cyrtodactylus makwanpurgadhiensis* sp. nov. (male, NHM 2025/383): A. Dorsal view of head; B. Ventral view of head; C. Lateral view of head on right; D. View of femoral region showing continuous series of precloacal pores; E. Ventral view of left manus, and F. Ventral view of left pes. Scale bars 5 mm; photographs by AK.

**Figure 5.**
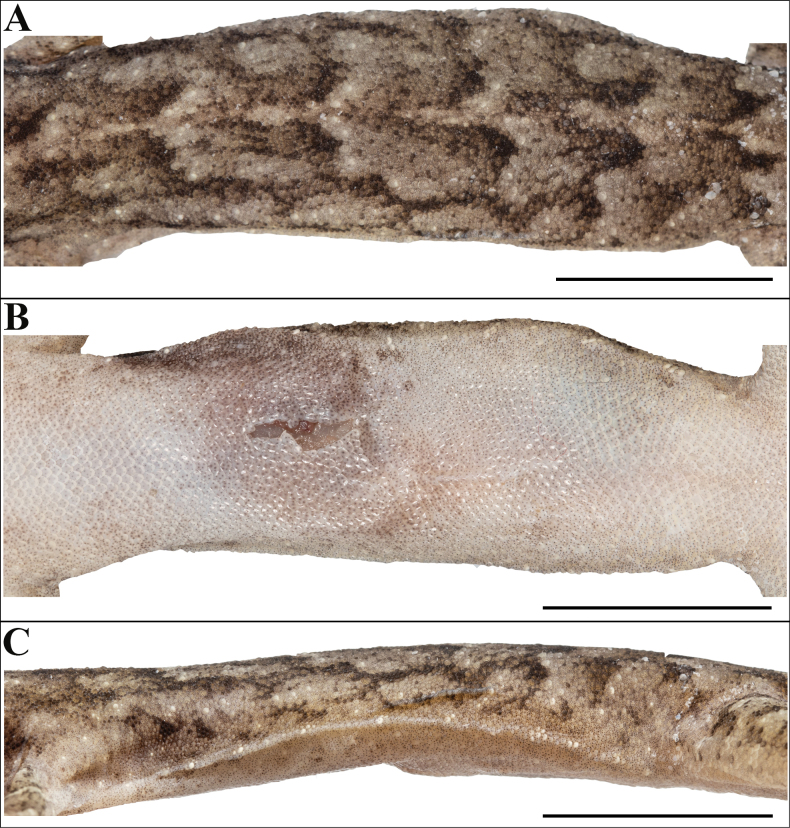
Holotype of *Cyrtodactylus makwanpurgadhiensis* sp. nov. (male, NHM 2025/383): A. Dorsal view of midbody; B. Ventral view of midbody, and C. Lateral view of midbody on right. Scale bars 10 mm; photographs by AK.

**Figure 6.**
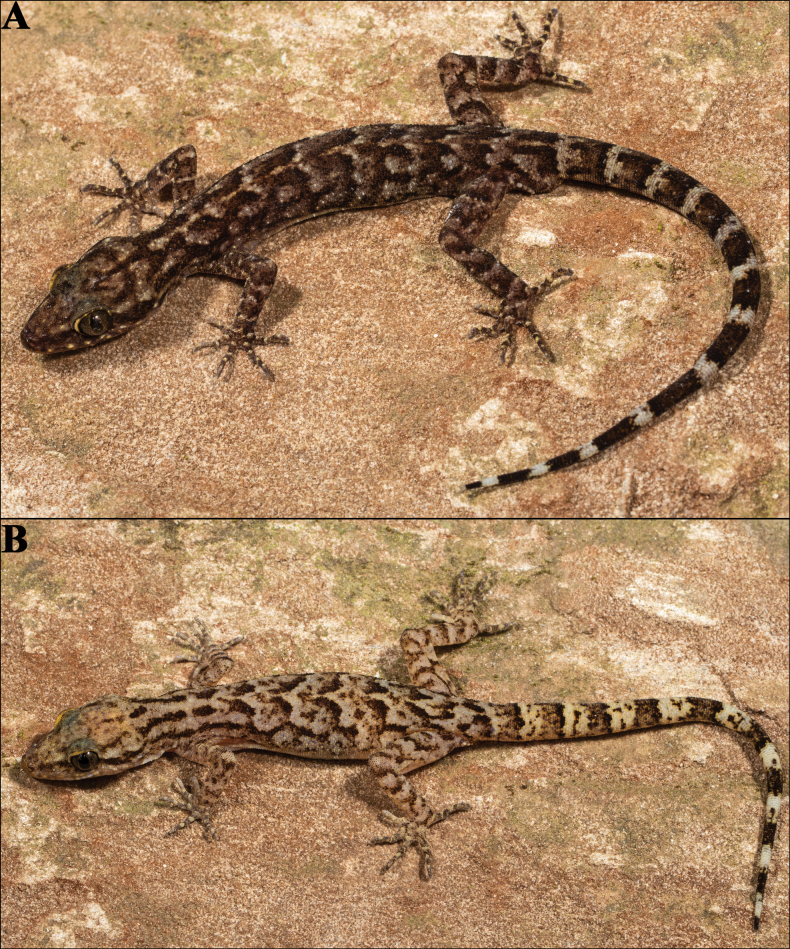
*Cyrtodactylus makwanpurgadhiensis* sp. nov., in life: A. Holotype (adult male, NHM 2025/383) and B. Paratype (adult female, NHM 2025/386). Photographs by AK.

**Figure 7.**
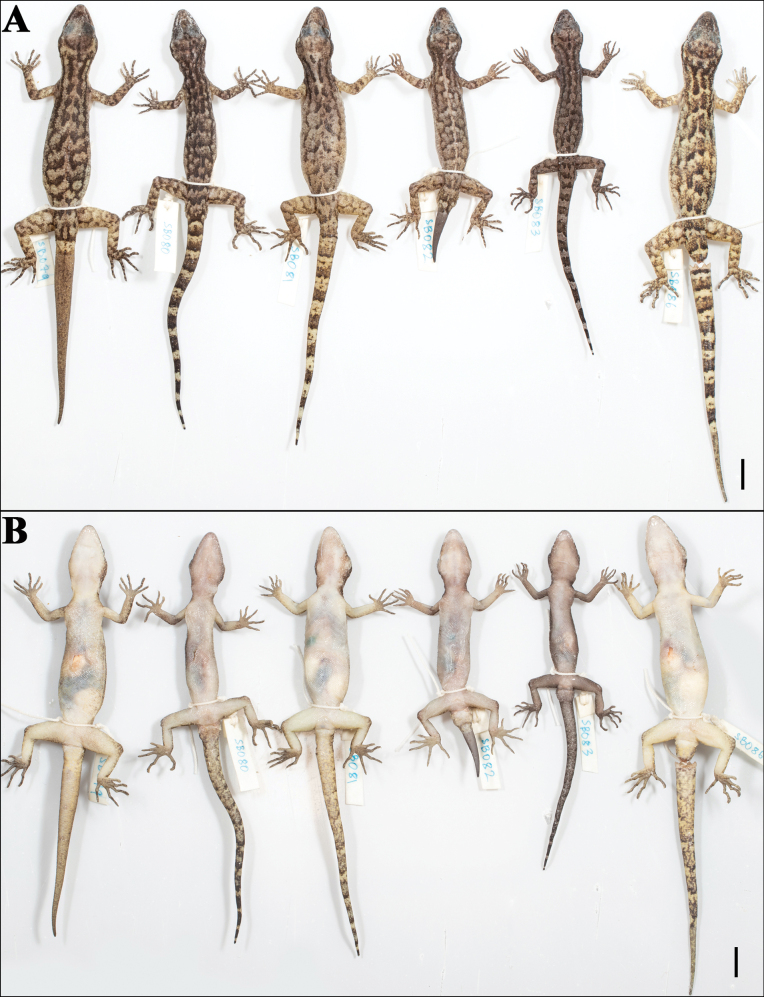
The paratype series of *Cyrtodactylus makwanpurgadhiensis* sp. nov., from left to right, NHM 2025/384–389: A. Dorsal view, and B. Ventral view. Scale bars 10 mm; photographs by AK.

Figs 3, 4, 5, 6, 7, Tables 2, 3

##### Type material examined.

***Holotype*.** • NHM 2025/383 (SB078), adult male, collected from on the walls of Makwanpurgadhi Fort (27°24.799'N, 85°8.690'E; ca 1050 m a.s.l.), Makwanpur District, Bagmati Province, Nepal; collected by Santosh Bhattarai on 10 July 2024. ***Paratypes*.** • NHM 2025/384 (SB079), NHM 2025/385 (SB080), NHM 2025/386 (SB081), NHM 2025/387 (SB082), NHM 2025/388 (SB083), NHM 2025/389 (SB086) bear the same locality and collection data as holotype.

##### Diagnosis.

A medium- sized *Cyrtodactylus*, snout to vent length up to 78.7 mm. Dorsal pholidosis heterogeneous; smooth granular scales intermixed with fairly regularly arranged rows of enlarged, feebly keeled, weakly pointed tubercles; a weak ventrolateral fold on lower flank; 18–20 rows of dorsal tubercles at midbody, 32–40 tubercles in paravertebral rows; ventral scales subequal from chest to vent, smooth, subcircular, and subimbricate with rounded end; 38–41 scales across belly at midbody, 76–90 longitudinal scales between axilla to groin, 167–195 longitudinal scales from mental to cloaca; subdigital scansors smooth, unnotched, and mostly entire; 12–14 lamellae under digit I of manus and pes, 16–18 lamellae under digit IV of manus and 19–23 lamellae under digit IV of pes; a series of nine precloacal pore-bearing scales contiguous with 10 or 11 enlarged precloacal scales in males (*n* = 4); female lack pores but have 7–9 pitted homologous scales, and 11–13 enlarged precloacal scales (*n* = 3); dorsal scales on non-regenerated tail homogeneous, fairly regularly arranged, smooth, subcircular, flattened, and subimbricate, and larger than granular scales at dorsal midbody, gradually becoming larger posteriorly and dorsolaterally; a few scattered enlarged tubercles present on the tail base; subcaudal scales in median series smooth, variable in size and shape, and not enlarged; variegated dorsal pattern, original tail bearing 10–13 alternating dark and light bands.

##### Genetic divergence.

*Cyrtodactylus makwanpurgadhiensis* sp. nov. is nested within the Nepalese clade of the Mountain subclade of the *khasiensis* group. It differs from members of the Nepalese clade by ≥ 14.5% uncorrected ND2 sequence divergence (Table 1).

##### Comparisons with regional congeners.

*Cyrtodactylus makwanpurgadhiensis* sp. nov. can be differentiated from all regional congeners based on the following differing or non-overlapping characters: no femoral pores and nine precloacal pores in males (vs femoral pores present in *C. chitwanensis*, *C. fasciolatus*, *C. gubernatoris*, and *C. nepalensis*; three or four precloacal pores in *C. annapurnaensis*, 6–9 in *C. cayuensis*, five in *C. chamba*, 10 in *C. himalayicus*, 7–11 in *C. kamengensis*, 4–9 in *C. lawderanus*, seven or eight in *C. martinstolli*; seven or eight precloacal pores and one or two pores below precloacal row in *C. karanshahi*); length of original tail > SVL (vs length of original tail < SVL in *C. lawderanus*); median row of subcaudals not enlarged (vs median row of subcaudals enlarged in *C. chitwanensis*, *C. fasciolatus*, and *C. nepalensis*); 18–20 rows of dorsal tubercles at midbody and 38–41 scales across belly at midbody (vs 24 or 25 DTR and 37 or 38 MVSR in *C. bhupathyi*, 13–15 DTR in *C. chamba*, 39–42 MVSR in *C. karanshahi*, 20–24 DTR and 30–34 MVSR in *C. kamengensis*, 19–23 DTR in *C. martinstolli*, 17 DTR in *C. nepalensis*, 15 or 16 DTR and 40–45 MVSR in *C. siangensis*); and moderate body size with maximum SVL up to 78.7 mm (vs maximum SVL < 65 mm in *C. annapurnaensis*, *C. bhupathyi*, *C. chamba*, *C. himalayicus* and maximum SVL > 80 mm in *C. cayuensis*, *C. chitwanensis*, *C. fasciolatus*, and *C. martinstolli*); 32–40 tubercles in paravertebral rows (vs 49–58 PVT in *C. kamengensis*); 12–14 lamellae under digit I of manus and pes, 16–18 lamellae under digit IV of manus (vs. 14 or 15 under digit I of manus and pes and 18–20 under digit IV of manus in *C. martinstolli*). *Cyrtodactylus makwanpurgadhiensis* sp. nov. is distinguished from the other new species described in this paper as part of its description below.

##### Description of the holotype.

Adult male in good state of preservation except tail bent towards left, and a 4.0 mm long incision in sternal region for tissue collection (Fig. 3A–D). SVL 66.8 mm, head short (HL/SVL 0.24), wide (HW/HL 0.69), not strongly depressed (HD/HL 0.38), distinct from neck. Loreal region inflated, canthus rostralis indistinct. Snout half of head length (ES/HL 0.44), twice the eye diameter (ES/ED 2.00); scales on snout and canthus rostralis oval, subequal, smooth, much larger than those on forehead and interorbital region; scales on forehead similar to those on snout and canthus rostralis except slightly smaller; scales on interorbital, occipital, and temporal regions heterogeneous, composed of granular scales intermixed with enlarged, feebly keeled, rounded tubercles (Fig. 4A). Eye small (ED/HL 0.22), with vertical pupil having crenulated margins; supraciliaries short, larger anteriorly; 15 interorbital scale rows across narrowest point of frontal; 47 scale rows between left and right supraciliaries at mid-orbit (Fig. 4A, C). Ear opening small, oval, deep (EL/HL 0.10); eye to ear distance much greater than diameter of eye (EE/ED 1.36) (Fig. 4C). Rostral ~2 × wider (2.8 mm) than high (1.5 mm), incompletely divided dorsally by a strongly developed rostral groove for ~1/2 of its height; a single enlarged, roughly rectangular supranasal on each side, > 5–6 × the size of upper postnasal, separated from each other behind rostral by a single much smaller internasal scale; rostral in contact with supralabial I, nostril and supranasal, and internasal on either side; nostrils oval, surrounded by three postnasals, supranasal, rostral, and supralabial I on either side; three subequal postnasals on either side; two rows of scales separate orbit from supralabials (Fig. 4C). Mental enlarged, subtriangular, slightly wider (2.8 mm) than high (2.4 mm); two pairs of postmentals, inner pair roughly triangular, slightly shorter (1.7 mm) than mental, in weak contact with each other below mental (0.3 mm); inner pair bordered by mental, infralabial I, outer postmental on either side and additionally by 11 slightly enlarged chin shields below; outer postmentals roughly rectangular, much smaller (0.8 mm) than inner pair, bordered by inner postmentals, infralabials I and II, and four chin shields on either side, 11 enlarged gular scales between left and right outer postmentals; all chin shields bordering postmentals flattened, subequal, subcircular, smooth, and much smaller than outermost postmentals; scales on rest of throat, granular, much smaller, smooth, and subcircular (Fig. 4B). Infralabials bordered below by a row or two of slightly enlarged, much elongated scales, decreasing in size posteriorly. Eleven supralabials to angle of jaw on either side and eight at midorbital position on left and seven on right side; supralabial I largest, gradually decreasing in size posteriorly; ten infralabials to angle of jaw on left and nine on right side, and seven at midorbital position on either side; infralabial I largest, gradually decreasing in size posteriorly (Fig. 4C).

Body relatively slender (BW/AGL 0.34), trunk slightly less than half of SVL (AGL/SVL 0.45) with weak ventrolateral fold (Fig. 5A–C). Dorsal pholidosis heterogeneous; smooth granular scales intermixed with fairly regularly arranged rows of enlarged, feebly keeled, weakly pointed tubercles; granular scales gradually increasing in size towards each flank, largest on mid-flank; granular scales on occiput slightly smaller than paravertebral granular scales; enlarged tubercles in ~19 longitudinal rows at midbody; 33 tubercles in paravertebral rows (Fig. 5A). Ventral scales much larger than granular scales on dorsum, subequal from chest to vent, smooth, subcircular and subimbricate with rounded end; scales on precloacal region distinctly enlarged; midbody scale rows across belly 41; 195 scales from mental to anterior border of cloaca and 87 scales between limb insertions (Fig. 5B). A continuous series of nine precloacal pores; femoral pores absent (Fig. 4D).

Scales on palm and soles, smooth, oval or subcircular, subequal and more or less flattened; scales on dorsal aspects of limbs heterogenous; composed of slightly smaller, smooth, subimbricate scales intermixed with enlarged, weakly keeled, weakly pointed tubercles which are slightly larger on thigh and shank than lower arm, enlarged tubercles absent on upper arm; scales on ventral aspect of upper arm smooth, granular, slightly smaller than granular scales on body dorsum, scales on ventral aspect of lower arm much larger than those on upper arm, smooth, subcircular, weakly conical to flattened, and subimbricate; ventral aspect of thigh and shank with enlarged, smooth, roughly rounded, flattened, subimbricate scales, slightly larger and oval on the shank but otherwise similar in size to those on body ventrals (Fig. 3A, B). Forelimbs and hindlimbs slightly long, slender (LAL/ SVL 0.14; CL/SVL 0.17); digits long, with a strong, recurved claw, distinctly inflected, distal portions laterally compressed conspicuously. Digits with mostly unpaired lamellae, separated into a basal and narrower distal series by a single, much enlarged lamella at inflection; basal lamellae series: (5-6-6-6-6 right manus, 3-6-7-8-7 right pes), (5-6-5-6-6 left manus, Fig. 4E; 3–6–7–9–6 left pes, Fig. 4F); distal lamellae series: (9-10-12-12-11 right manus, 10-11-13-12-14 right pes), (9-10-12-12-11 left manus, Fig. 4E; 10–11–14–13–13 left pes, Fig. 4F). Relative length of digits (measurements in mm in parentheses): IV (5.8) > III (5.4) > V (5.1) > II (4.9) > I (3.6) (left manus); IV (7.3) > III (6.9) > V (6.3) > II (5.4) > I (3.6) (left pes).

Tail original, subcylindrical, slender, entire, slightly longer than body (TL/SVL 1.20) (Fig. 3C, D). Dorsal pholidosis on tail homogeneous; composed of fairly regularly arranged, smooth, subcircular, flattened, and subimbricate scales that are larger than granular scales on midbody dorsum, gradually becoming larger posteriorly and dorsolaterally; a few scattered enlarged tubercles present on the tail base (Fig. 3C). Scales on tail venter much larger than those on dorsal aspect, smooth, flattened, subimbricate; median series smooth, variable in size and shape, and not enlarged (Fig. 3D). Scales on tail base much smaller, smooth, subimbricate; three subequal and smooth postcloacal tubercles on left and four on right side (Fig. 3D).

##### Colouration in life

**(Fig. 6A).** Dorsal ground colour of head, body, limbs and tail pale brown, strongly variegated with thick dark brown or black reticulations and pale brown blotches; labials with a few yellow streaks; distinct dark brown pre and postorbital streaks; a discontinuous light mid-vertebral stripe from neck to tail base; 12 dark and 13 pale caudal bands on original tail; rest of ventral surfaces immaculate; iris green-grey with dark reticulations, pupil bordered by pale orange.

##### Variation and additional information from paratypes

**(Figs 6B, 7).** Mensural, meristic, and additional character state data for the type series is given in Tables 2, 3 respectively. There are three adult females, two adult males, and a single subadult male ranging in size from 54.9–78.7 mm (Fig. 7A, B). All paratypes resemble the holotype except as follows: Five paratypes—NHM 2025/384, NHM 2025/385, NHM 2025/386, NHM 2025/387, and NHM 2025/388 with total three internasals, the one touching to rostral is ~3 × larger than the remaining two internasals. Inner postmentals bordered by mental, infralabial I, and outer postmental in all paratypes; additionally, bordered by six smaller chin shields in NHM 2025/384, eight in NHM 2025/385 and NHM 2025/389, 12 in NHM 2025/386, seven in NHM 2025/387, and nine in NHM 2025/388. Outer postmentals bordered by inner pair and infralabial I & II in all paratypes; additionally, bordered by four smaller chin shields on left and five on right side in NHM 2025/386 and NHM 2025/388, and by five on either side in NHM 2025/389. Three paratypes, NHM 2025/385, NHM 2025/386, and NHM 2025/388 with original and complete tail, marginally longer than SVL (TL/SVL 1.14, 1.12, and 1.14 respectively); NHM 2025/389 with complete but partially regenerated tail, which is detached from the body, marginally longer than body (TL/SVL 1.06); NHM 2025/384 with complete but fully regenerated tail, slightly shorter than body (TL/SVL 0.85); and NHM 2025/387 with incomplete regenerating tail; original tail distinctly banded and regenerated tail light brown (Fig. 7A, B).

**Table 2.** Mensural (mm) data for *Cyrtodactylus makwanpurgadhiensis* sp. nov., and *C. chure* sp. nov. Abbreviations are listed in Materials and Methods except for: M = male, F = female, SA = subadult male, and * = tail incomplete.

Species	*C. makwanpurgadhiensis* sp. nov.							*C. chure* sp. nov.			
Type	Holotype	Paratypes						Holotype	Paratypes		
Specimen Number	NHM 2025/383	NHM 2025/384	NHM 2025/385	NHM 2025/386	NHM 2025/387	NHM 2025/388	NHM 2025/389	NHM 2025/379	NHM 2025/380	NHM 2025/381	NHM 2025/382
Sex	M	F	M	F	M	SA M	F	M	M	F	F
SVL	66.8	75.6	66.2	69.3	61.8	54.9	78.7	60.0	58.4	68.7	63.8
TL	79.9	63.9	75.3	77.6	24.4*	62.6	83.7	66.6	63.7	48.6	67.5
TW	7.4	6.6	6.9	6.1	6.5	5.6	6.5	6.8	6.0	5.0	4.8
LAL	9.4	10.1	9.8	8.9	8.6	8.2	10.0	9.2	7.4	8.7	8.5
CL	11.1	12.7	11.4	11.3	10.9	9.4	12.7	10.6	10.1	11.7	10.8
AGL	30.1	35.2	29.9	33.6	30.1	26.5	37.2	26.4	25.6	29.7	29.5
BH	6.4	8.6	5.8	9.3	6.4	5.8	9.9	5.5	5.8	8.1	7.4
BW	10.2	14.7	10.3	14.3	10.3	8.5	14.7	11.0	10.2	14.4	13.3
HL	16.3	17..7	16.2	17.2	15.5	13.2	18.8	15.3	14.2	15.9	15.7
HW	11.2	12.2	11.4	11.6	10.6	10.2	12.9	10.7	9.9	12.0	10.8
HH	6.2	7.1	6.6	6.8	6.5	5.8	7.4	6.1	5.6	6.5	6.4
ED	3.6	4.0	3.4	3.7	3.5	2.7	4.4	4.1	3.7	3.6	3.8
EE	4.9	5.7	4.7	5.2	4.5	3.9	5.5	4.8	4.5	4.8	4.5
ES	7.2	8.3	7.0	7.5	6.8	6.3	8.7	6.8	6.6	7.6	7.3
EN	5.2	6.2	5.6	6.1	4.8	4.4	6.4	5.1	5.1	5.2	5.3
IN	2.1	2.2	1.9	2.0	1.9	1.5	2.5	2.2	1.9	2.0	2.0
IO	3.3	3.6	3.2	3.8	3.5	2.8	3.9	3.7	3.4	4.1	3.3
EL	1.6	2.2	1.9	1.5	1.8	1.5	1.8	1.5	1.2	1.7	1.6

**Table 3.** Meristic data for *Cyrtodactylus makwanpurgadhiensis* sp. nov. and *C. chure* sp. nov. The values in parentheses are the number of pitted scales in females. Abbreviations are listed in Materials and methods except for: M = male, F = female, SA = subadult, L&R = left & right, P/A = present/absent, * = incomplete count, / = data unavailable; numbers in parentheses for PCS indicates number of pitted scales in females.

Species	*C. makwanpurgadhiensis* sp. nov.							*C. chure* sp. nov.			
Type	Holotype	Paratypes						Holotype	Paratypes		
Specimen Number	NHM 2025/383	NHM 2025/384	NHM 2025/385	NHM 2025/386	NHM 2025/387	NHM 2025/388	NHM 2025/389	NHM 2025/379	NHM 2025/380	NHM 2025/381	NHM 2025/382
Sex	M	F	M	F	M	SAM	F	M	M	F	F
INS	1	3	3	3	3	3	1	3	1	1	3
SL L&R	11&11	12&12	11&12	11&12	11&11	11&11	13&12	12&12	11&11	11&11	13&12
IL L&R	10&9	9&9	10&10	9&9	10&11	9&10	9&9	10&11	10&9	11&10	11&11
SL M L&R	8&7	8&8	8&9	8&8	8&8	8&8	8&7	8&8	8&8	8&8	8&8
IL M L&R	7&7	6&6	6&6	7&7	7&8	7&7	6&6	6&6	6&8	7&7	7&7
PVT L&R	33&33	36&36	33&32	35&36	35&34	33&35	37&40	34&34	36&37	36&37	34&35
DTR	19	20	18	18	19	20	20	18	18	20	19
MVSR	41	39	39	40	38	38	39	38	38	37	38
VS1	87	82	85	76	90	76	79	80	/	79	86
VS2	195	173	180	167	179	170	175	177	/	167	184
DLAMF1 L&R	9&9	9&8	7&8	8&8	8&8	8&8	8&8	8&8	7&8	9&9	8&8
BLAMF1 L&R	5&5	5&5	5&5	5&5	5&5	4&4	5&5	5&5	6&5	5&5	4&5
DLAMF4 L&R	12&12	11&11	11&12	11&11	12&13	11&12	11&10	12&12	10&11	12&12	11&11
BLAMF4 L&R	6&6	6&6	7&6	6&6	6&5	6&6	6&6	6&6	6&6	6&6	6&5
DLAMT1 L&R	10&10	9&9	8&8	9&9	9&10	8&8	8&8	9&9	9&9	9&9	9&9
BLAMT1 L&R	3&3	4&4	4&4	4&4	4&4	4&4	4&5	3&3	4&4	4&4	5&5
DLAMT4 L&R	13&12	13&13	12&14	13&13	14&13	13&12	11&12	12&12	12&12	13&13	12&13
BLAMT4 L&R	9&8	8&9	9&9	7&7	9&6	8&8	8&8	6&6	6&6	6&6	8&8
DLAMT5 L&R	13&14	13&13	13&12	13&12	13&13	12&13	12&12	13&12	11&11	14&12	12&12
BLAMT5 L&R	6&7	6&7	6&6	5&6	6&6	6&6	6&7	5&6	6&6	5&5	6&6
TLAMF1 L&R	14&14	14&13	12&13	13&13	13&13	12&12	13&13	13&13	13&13	14&14	14&14
TLAMF4 L&R	18&18	17&17	18&18	17&17	18&18	17&18	17&16	18&18	16&17	18&18	17&16
TLAMT1 L&R	13&13	13&13	12&12	13&13	13&14	12&12	12&13	12&12	13&13	13&13	14&14
TLAMT4 L&R	22&20	21&22	21&23	20&20	23&19	21&20	19&20	18&18	18&18	19&19	20&21
TLAMT5 L&R	19&21	19&20	19&18	18&18	19&19	18&19	18&19	18&18	17&17	19&17	18&18
PCS	11	13 (9)	10	11 (9)	11	11	11 (7)	9	10	12 (8)	9 (5)
PP L&R	9	A	9	A	9	9	A	7	8	A	A
PCT L&R	3&4	3&3	3&3	3&3	4&4	3&3	3&3	3&3	4&4	3&3	2&3
Caudal tubercles P/A	A	/	A	A	/	A	A	A	A	/	A
Subcaudals enlarged or not	NOT EN	/	NOT EN	NOT EN	/	NOT EN	NOT EN	NOT EN	NOT EN	/	NOT EN

##### Etymology.

The specific epithet is a toponym for Makwanpurgadhi (Gadhi = Fort), which is ~17 km north-east of Hetauda town, Makwanpur District in Bagmati Province. Makwanpurgadhi is the largest fort in Nepal and was established in the 16^th^ century during the Sen dynasty. Suggested common name is Makwanpurgadhi bent-toed gecko.

##### Distribution and natural history.

**Figure 8.**
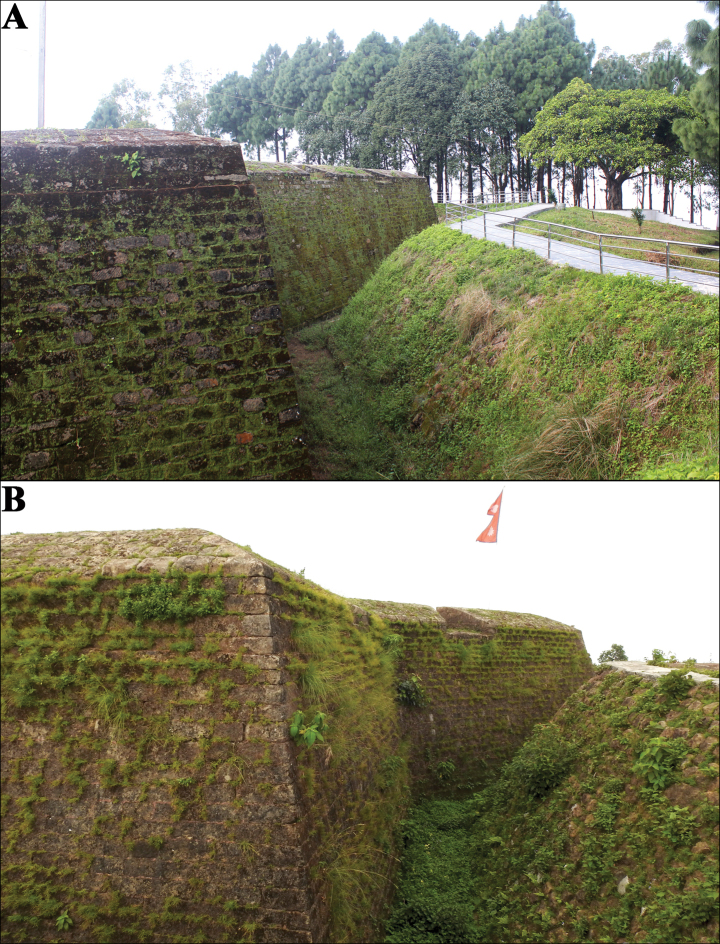
Habitat photos at the type localities of the two new species: A. *Cyrtodactylus makwanpurgadhiensis* sp. nov. and B. *Cyrtodactylus chure* sp. nov. showing their micro habitats. Photographs (A) by BN and (B) by BG.

We spotted ~20–25 individuals from ca 1930–2130 hrs on 10 July 2024 between the heights of < 10 cm to ~5 m on the walls of Makwanpurgadhi Fort and along roadside walls between Makwanpurgadhi and Hetauda town (Fig. 1). The walls of the fort were partially covered with algae and had numerous crevices (Fig. 8A). Broadly within the sub-tropical Sal mixed forest belt, there is little natural vegetation around the fort as it is a popular tourist destination with manicured lawns by day, but relatively quiet and calm at night. No disturbances were observed during nighttime sampling, although individuals facing the road are likely to have to contend with lights from passing traffic. We did not sample any forest areas. Other lizards observed here were *Calotes versicolor* (Daudin, 1802) and *Hemidactylus* sp.

#### ﻿ Cyrtodactylus chure sp. nov.

Taxon classificationAnimaliaSquamataGekkonidae

9FBED2B1-0283-548F-B367-379B21E149F0

https://zoobank.org/ED853DDF-2277-44D2-8F4A-ADB1CC124D6A

**Figure 9.**
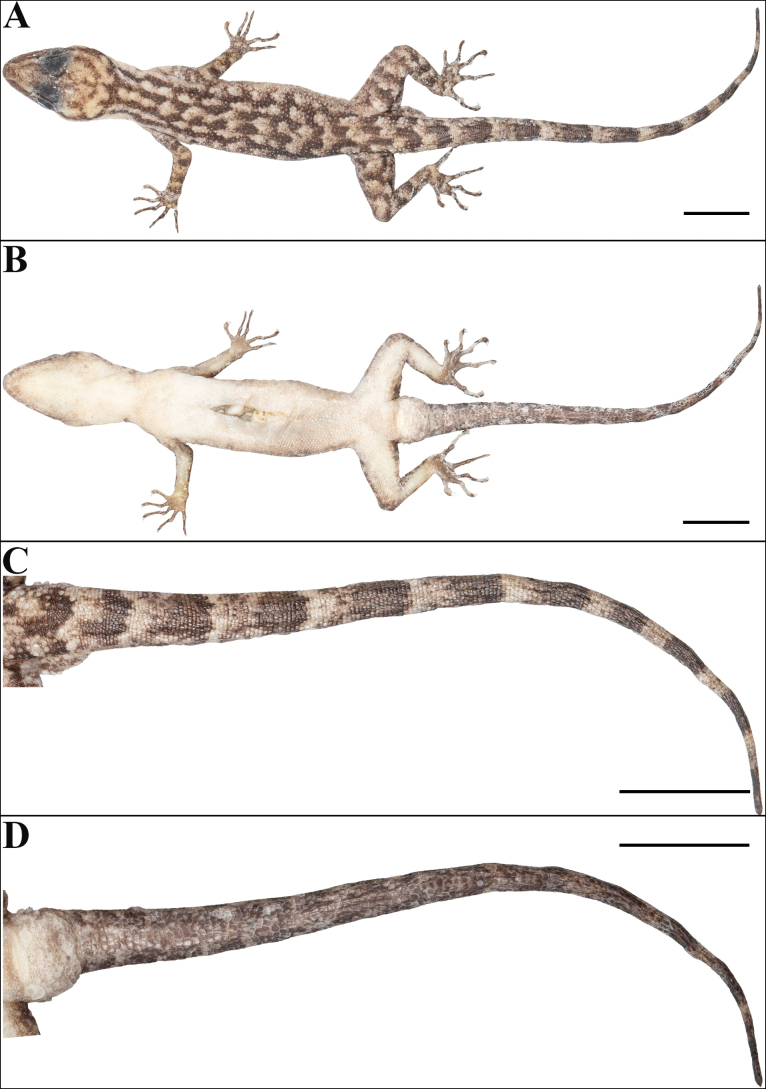
Holotype of *Cyrtodactylus chure* sp. nov. (male, NHM 2025/379): A. Dorsal view of body; B. Ventral view of body; C. Dorsal view of tail, and D. Ventral view of tail. Scale bars 10 mm; photographs by AK.

**Figure 10.**
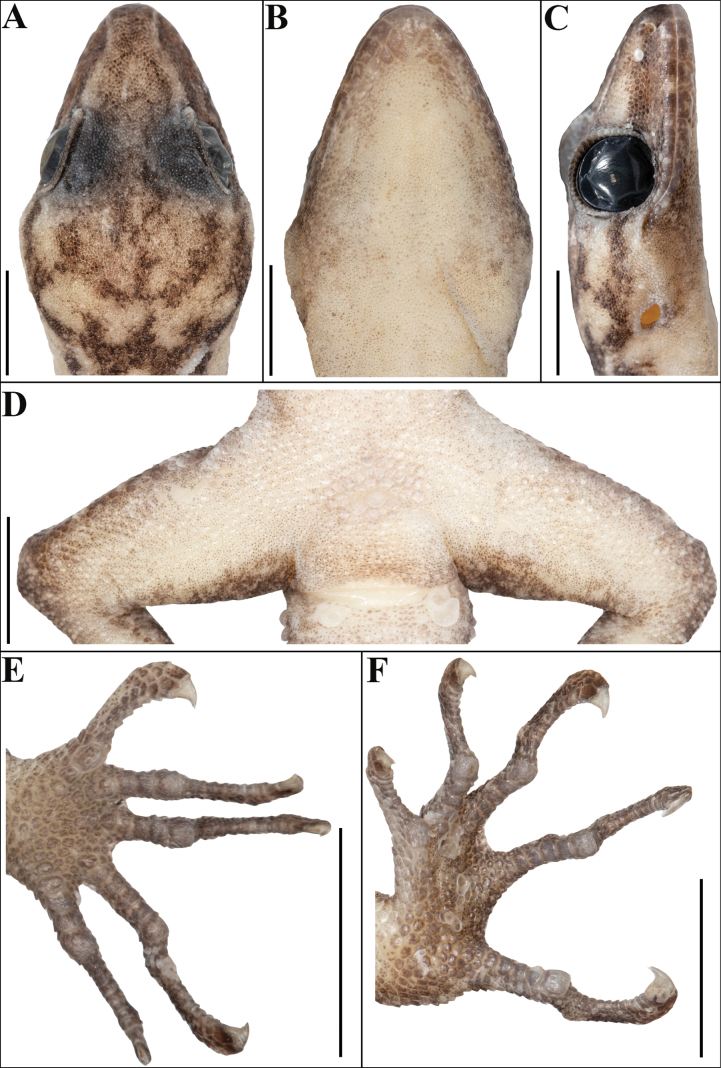
Holotype of *Cyrtodactylus chure* sp. nov. (male, NHM 2025/379): A. Dorsal view of head; B. Ventral view of head; C. Lateral view of head on right; D. View of femoral region showing continuous series of precloacal pores; E. Ventral view of left manus, and F. Ventral view of left pes. Scale bars 5 mm; photographs by AK.

**Figure 11.**
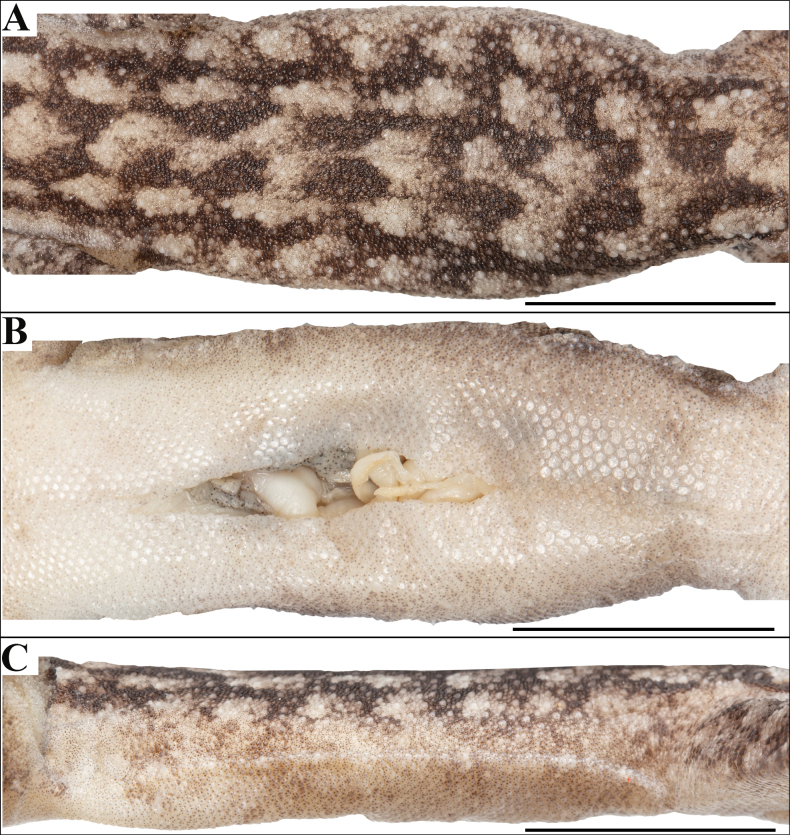
Holotype of *Cyrtodactylus chure* sp. nov. (male, NHM 2025/379): A. Dorsal view of midbody; B. Ventral view of midbody, and C. Lateral view of midbody on right. Scale bars 10 mm; photographs by AK.

**Figure 12.**
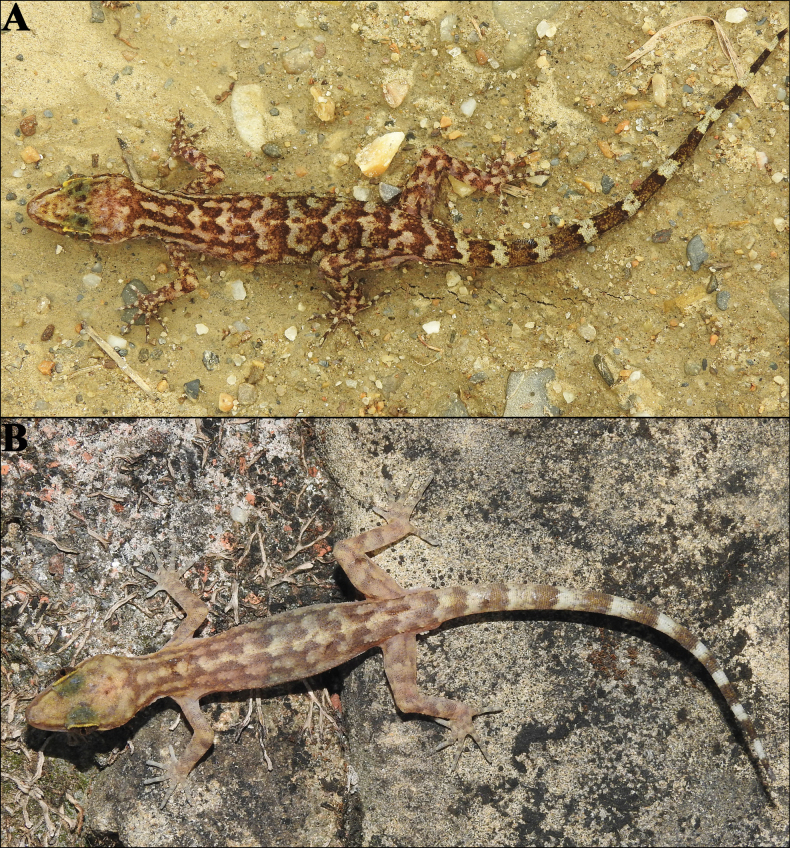
*Cyrtodactylus chure* sp. nov., in life: A. Holotype (adult male, NHM 2025/379) and B. Paratype (adult male, NHM 2025/382). Photographs by BG.

**Figure 13.**
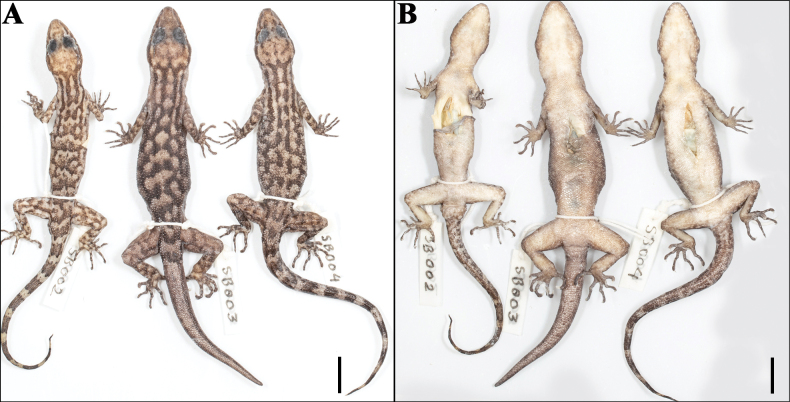
The paratype series of *Cyrtodactylus chure* sp. nov., from left to right, NHM 2025/380–382: A. Dorsal view, and B. Ventral view. Scale bars 10 mm; photographs by AK.

Figs 9, 10, 11, 12, 13, Tables 2, 3

##### Type material examined.

***Holotype*.** • NHM 2025/379 (SB001), adult male, collected from on the walls of Hariharpurgadhi Fort (27°18.820'N, 85°29.223'>E; ca 905 m a.s.l.), Sindhuli District, Bagmati Province, Nepal; collected by Santosh Bhattarai on 16 June 2024. ***Paratypes*.** • NHM 2025/380 (SB002), NHM 2025/381 (SB003), NHM 2025/382 (SB004) bears the same locality and collection data as holotype.

##### Diagnosis.

A medium-sized *Cyrtodactylus*, snout to vent length up to 68.7 mm. Dorsal pholidosis heterogeneous; smooth granular scales intermixed with fairly regularly arranged rows of enlarged, feebly keeled, weakly pointed tubercles; a weak ventrolateral fold on lower flank; 18–20 rows of dorsal tubercles at midbody, 34–37 tubercles in paravertebral rows; ventral scales subequal from chest to vent, smooth, subcircular, and subimbricate with rounded end; 37 or 38 scales across belly at midbody, 79–86 longitudinal scales between axilla to groin, 167–184 longitudinal scales from mental to cloaca; subdigital scansors smooth, unnotched, and mostly entire; 14 or 13 lamellae under digit I of manus and 12–14 lamellae under digit I of pes, 16–18 lamellae under digit IV of manus and 18–21 lamellae under digit IV of pes; a series of seven or eight precloacal pore-bearing scales contiguous with nine or ten enlarged precloacal scales in males (*n* = 2); females lack pores but have 5–8 pitted homologous scales, and 9–12 enlarged precloacal scales (*n* = 2); dorsal scales on non-regenerated tail homogeneous, fairly regularly arranged, smooth, elongated, flattened, subimbricate, and larger than granular scales at dorsal midbody, gradually becoming larger posteriorly and dorsolaterally; a few scattered enlarged tubercles present on the tail base; subcaudal scales in median series smooth, variable in size and shape, and not enlarged; dorsal pattern of ~9 dark-brown, broken cross-bars, original tail bearing ten or 11 alternating dark and lighter bands.

##### Genetic divergence.

*Cyrtodactylus chure* sp. nov. is nested within the Nepalese clade within the Mountain subclade of the *khasiensis* group. It differs from members of the Nepalese clade by ≥ 11.2% uncorrected ND2 sequence divergence (Table 1).

##### Comparisons with regional congeners.

*Cyrtodactylus chure* sp. nov. can be differentiated from all regional congeners based on the following differing or non-overlapping characters: no femoral pores and seven or eight precloacal pores in males (vs femoral pores present in *C. chitwanensis*, *C. fasciolatus*, *C. gubernatoris*, and *C. nepalensis*; three or four precloacal pores in *C. annapurnaensis*, 6–9 in *C. cayuensis*, five in *C. chamba*, 10 in *C. himalayicus*, 7–11 in *C. kamengensis*, nine in *C. makwanpurgadhiensis* sp. nov.; seven or eight precloacal pores and one or two pores below precloacal row in *C. karanshahi*); length of original tail > SVL (vs length of original tail < SVL in *C. lawderanus*); median row of subcaudals not enlarged (vs median row of subcaudals enlarged in *C. chitwanensis*, *C. fasciolatus*, *C. nepalensis*); 18–20 rows of dorsal tubercles at midbody and 37 or 38 scales across belly at midbody (vs 24 or 25 DTR in *C. bhupathyi*, 13–15 DTR in *C. chamba*, 20–24 DTR and 30–34 MVSR in *C. kamengensis*, 39–42 MVSR in *C. karanshahi*, 19–23 DTR in *C. martinstolli*, 17 DTR in *C. nepalensis*, 15 or 16 DTR and 40–45 MVSR in *C. siangensis*); 16–18 lamellae under digit IV of pes (vs 19–22 in *C. martinstolli*); and maximum SVL up to 68.7 mm (vs maximum SVL < 65 mm in *C. annapurnaensis*, *C. bhupathyi*, *C. chamba*, *C. himalayicus* and maximum SVL > 78 mm in *C. cayuensis*, *C. chitwanensis*, *C. fasciolatus*, *C. kamengensis*, *C. makwanpurgadhiensis* sp. nov., *C. martinstolli*) 34–37 tubercles in paravertebral rows (vs 49–58 PVT in *C. kamengensis*). *Cyrtodactylus chure* sp. nov. overlaps with *C. makwanpurgadhiensis* sp. nov. in all meristic data except for 16–18 lamellae under digit IV of pes (vs 19–23 in *C. makwanpurgadhiensis* sp. nov.), and can be distinguished by a slightly shorter body (mean (minimum–maximum) AGL/ SVL = 0.443 (0.432–0.462) vs 0.469 (0.451–0.487) and slightly longer crus (CL/ SVL = 0.168 (0.161–0.176) vs 0.172 (0.169–0.177).

##### Description of the holotype.

Adult male in good state of preservation except tail bent towards left, and a 12.3 mm long incision in sternal region for tissue collection (Fig. 9A–D). SVL 60.0 mm, head short (HL/SVL 0.26), wide (HW/HL 0.70), not strongly depressed (HD/HL 0.40), distinct from neck. Loreal region inflated, canthus rostralis indistinct. Snout half of head length (ES/HL 0.44), slightly > 1.5 × eye diameter (ES/ED 1.66); scales on snout and canthus rostralis circular or oval, subequal, smooth, much larger than those on forehead and interorbital region; scales on forehead similar to those on snout and canthus rostralis except slightly smaller; scales on interorbital region, occipital, and temporal region heterogeneous, composed of granular scales intermixed with enlarged, smooth, and rounded tubercles (Fig. 10A). Eye small (ED/HL 0.27), with vertical pupil having crenulated margins; supraciliaries short, larger anteriorly; 17 interorbital scale rows across narrowest point of frontal; 39 scale rows between left and right supraciliaries at mid-orbit (Fig. 10A, C). Ear opening small, oval, deep (EL/ HL 0.10); eye to ear distance slightly greater than diameter of eye (EE/ED 1.17) (Fig. 10C). Rostral ~2 × wider (2.4 mm) than high (1.4 mm), incompletely divided dorsally by a strongly developed rostral groove for slightly less than half of its height; a single enlarged, roughly circular supranasal on each side, > 4-5 × the size of upper postnasal, separated from each other behind rostral by three much smaller internasal scales; rostral in contact with supralabial I, nostril and supranasal, and single internasal on either side; nostrils oval, surrounded by three postnasals, supranasal, rostral, and supralabial I on either side; three postnasals on either side, middle postnasal roughly oval, slightly larger than others; other postnasals roughly circular and subequal; two rows of scales separate orbit from supralabials (Fig. 10C). Mental enlarged, subtriangular, wider (2.3 mm) than high (1.6 mm); two pairs of postmentals, inner pair rectangular, marginally longer (1.8 mm) than mental, in strong contact with each other below mental (0.9 mm); inner pair bordered by mental, infralabial I, outer postmental, and two slightly enlarged chin shields on left and three on right side; outer postmentals roughly rectangular, much smaller (0.8 mm) than inner pair, bordered by inner postmentals on either side, infralabials I and II on right and II on left side, and four chin shields on right and six on left side, five enlarged gular scales between left and right outer postmentals; all chin shields bordering postmentals somewhat protruding, subequal, subcircular, smooth, and much smaller than outermost postmentals; scales on rest of throat, granular, much smaller, smooth, and subcircular (Fig. 10B). Infralabials bordered below by a row or two of slightly enlarged, much elongated scales, decreasing in size posteriorly. Eleven supralabials to angle of jaw and eight at midorbital position on either side; supralabial I largest, gradually decreasing in size posteriorly; ten infralabials to angle of jaw on left and nine on right side, and six infralabials at midorbital position on left and eight on right side; infralabial I largest, gradually decreasing in size posteriorly (Fig. 10C).

Body relatively slender (BW/AGL 0.42), trunk slightly less than half of SVL (AGL/SVL 0.44) with weak ventrolateral fold (Fig. 11A–C). Dorsal pholidosis heterogeneous; smooth granular scales intermixed with fairly regularly arranged rows of enlarged, feebly keeled, weakly pointed tubercles; granular scales gradually increasing in size towards each flank, largest on mid-flank; granular scales on occiput slightly smaller than paravertebral granular scales; enlarged tubercles in ~18 longitudinal rows at midbody; 34 tubercles in paravertebral rows (Fig. 11A). Ventral scales much larger than granular scales on dorsum, subequal from chest to vent, and smooth, subcircular and subimbricate with rounded end; scales on precloacal region distinctly enlarged; midbody scale rows across belly 38; 177 scales from mental to anterior border of cloaca and 80 scales between limb insertions (Fig. 11B). A continuous series of seven precloacal pores, femoral pores absent (Fig. 10D).

Scales on palm and soles, smooth, oval or rounded, and flattened; scales on dorsal aspects of limbs heterogenous; composed of slightly smaller, smooth granular scales intermixed with enlarged, weakly keeled, weakly pointed tubercles which are slightly larger on thigh and shank than lower arm, enlarged tubercles absent on the upper arm; scales on ventral aspect of upper arm smooth, granular, slightly smaller than granular scales on body dorsum, scales on ventral aspect of lower arm much larger than those on upper arm, smooth, subcircular, weakly conical to flattened, and subimbricate; ventral aspect of thigh and shank with enlarged, smooth, roughly rounded, flattened, subimbricate scales, slightly larger and oval on the shank but otherwise similar in size to those on body ventrals (Fig. 9A, B). Forelimbs and hindlimbs slightly long, slender (LAL/ SVL 0.15; CL/SVL 0.18); digits long, with a strong, recurved claw, distinctly inflected, distal portions laterally compressed conspicuously. Digits with mostly unpaired lamellae, separated into a basal and narrower distal series by a single, much enlarged lamella at inflection; basal lamellae series: (5-6-6-6-5 right manus, 3-6-7-6-6 right pes), (5-6-6-6-5 left manus, Fig. 10E; 3–6–7–6–5 left pes, Fig. 10F); distal lamellae series: (8-9-12-12-10 right manus, 9-10-12-12-12 right pes), (8-10-11-12-9 left manus, Fig. 10E; 9–10–12–12–13 left pes, Fig. 10F). Relative length of digits (measurements in mm in parentheses): IV (4.6) = III (4.6) > V (4.2) > II (3.9) > I (2.8) (left manus); IV (5.5) > V (5.4) > III (5.3) > II (4.2) > I (2.8) (left pes).

Tail original, subcylindrical, slender, entire, marginally longer than body (TL/SVL 1.11) (Fig. 9C, D). Dorsal pholidosis on tail homogeneous; composed of fairly regularly arranged, smooth, elongated, flattened, and subimbricate scales that are larger than granular scales on midbody dorsum, gradually becoming larger posteriorly and dorsolaterally; a few scattered enlarged tubercles present on the tail base (Fig. 9C). Scales on tail venter much larger than those on dorsal aspect, smooth, flattened, subimbricate; median series smooth, variable in size and shape, and not enlarged (Fig. 9D). Scales on tail base much smaller, smooth, subimbricate; three subequal and smooth postcloacal tubercles on either side (Fig. 9D).

##### Colouration in life

**(Fig. 12).** Dorsal ground colour of head, body, limbs and tail brown; labials slightly darker than top of head and with a few yellow streaks; distinct dark brown pre- and postorbital streaks; no mid-vertebral stripe; ~9 broken up dark brown transverse markings from neck to tail-base; 11 dark and ten light caudal bands on original tail; rest of ventral surfaces immaculate; iris bronze with dark reticulations, pupil bordered by pale orange.

##### Variation and additional information from paratypes

**(Figs 12, 13).** Mensural and meristic data for the type series is given in Tables 2, 3, respectively. There are two adult females and a single adult male ranging in size from 58.4–68.7 mm (Fig. 13A, B). All paratypes resemble the holotype except as follows: NHM 2025/380 and NHM 2025/381 with single internasal separating supranasals behind rostral. Inner postmentals bordered by mental, infralabial I, and outer postmental; additionally, bordered by six smaller chin shields in all paratypes. Outer postmentals bordered by inner pair and infralabials I and II in all paratypes except for NHM 2025/381; additionally, bordered by five smaller chin shields on left and four on right side either in NHM 2025/380, by five on either side in NHM 2025/382; outer postmentals bordered by inner pair, infralabial I, and four smaller chin shields on either side in NHM 2025/381. Two paratypes, NHM 2025/380 and NHM 2025/382 with original and complete tail, marginally longer than SVL (TL/SVL 1.06 and 1.09 respectively); NHM 2025/381 with complete but almost fully regenerated tail, slightly shorter than body (TL/SVL 0.70) (Fig. 13A). Original tail distinctly banded in all paratypes and regenerated tail pale brown in NHM 2025/381 (Figs 12, 13).

##### Etymology.

The specific epithet, Chure (ch-oo-ray), is the Nepali word for the Siwalik Mountain range, within which the type locality lies, and is used as a noun in apposition. In Nepal, Chure is widely used among policy makers, conservationists, and local communities to refer to the Siwaliks. These are the youngest, driest, least geologically stable, and southernmost of the Himalayan ranges, delineating the boundary with the lowland (Terai) plains. Suggested common name is Chure bent-toed gecko.

##### Distribution and natural history.

Individuals were found ca 1930–2330 hrs on 16 June 2024 on the walls of Hariharpurgadhi Fort in Sindhuli District, Bagmati Province, Nepal (Fig. 1), which was constructed in mid-16^th^ century during Sen dynasty. Two to three individuals were observed within ~1 m on the fort wall ~1–3 m above ground level. The walls of the fort were shaded in patches by bushes and low-growing grasses and had numerous crevices (Fig. 8B). There is little natural vegetation around the fort and we did not sample any forest patches. The fort is relatively remote, located within the Siwalik Mountain range ca 40 km away from the nearest township (Sindhuli Madi). There is little human visitation and, currently, low levels of vehicle traffic. However, road construction projects across the Siwalik mountains, including a road development between Sindhuli and Makwanpur, will increase the volume of traffic in the near future. Currently, the fort area during the night is calm and the animals appeared sensitive to flashlights. Other lizards observed were *Hemidactylus platyurus* (Schneider, 1797) at night and *Eutropis carinata* (Schneider, 1801) during the day.

## ﻿Discussion

Only five species of *Cyrtodactylus* have been described from Nepal prior to this study, indicating that herpetological surveys in the region have been limited. Yet Central Nepal is considered to support a particularly high diversity of herpetofauna because of its highly variable geography and climate (Schleich and Kästle 2002; Shah and Tiwari 2004; Bhattarai et al. 2020). The unique geographical position of central Nepal between the western Himalaya (west of Kali Gandaki River) and the eastern Himalaya (east of Teesta River) serves as a transitional zone with highly variable rainfall, temperature seasonality and vegetation types (Mani 1974; Paudel et al. 2012). This environmental and biogeographical heterogeneity is likely to support several undiscovered species in the Nepal Himalaya. Habitats suitable for *Cyrtodactylus* are known to occur in Nepal (Bhattarai et al. 2025; Grismer et al. 2020), which is one of the 200 global priority ecoregions (Olson et al. 2001; Olson and Dinerstein 2002). While the central Himalayas are often described as a dispersal barrier for species diversification and distribution (Manish and Pandit 2018), the area is also characterised by the presence of species whose origins are from the east and west. For this reason, it is considered an area of faunal exchange. *Cyrtodactylus* supports this hypothesis by the presence of the westernmost representatives of the mountain subclade of the *khasiensis* group and the easternmost representatives of the *fasciolatus* group in central Nepal.

Of the five species of *Cyrtodactylus* from central Nepal; two species, *C. annapurnaensis* and *C. karanshahi*, are reported from locations within Nepal’s system of protected areas and the remaining three, namely *C. chitwanensis*, *C. makwanpurgadhiensis* sp. nov. and *C. chure* sp. nov. occur outside protected areas (Fig. 1). A global assessment by Cox et al. (2022) revealed that one of every five reptile species are facing extinction risks, and that this risk is highest for reptiles occurring outside of protected areas. In general, the focus of Nepal’s conservation and research is directed towards large mammals and charismatic species and most studies are located within the Protected Area Network (Bist et al. 2021). Lesser-known species such as reptiles are overlooked (Rawat et al. 2020) which is further compounded for species that occur outside protected areas (Gautam et al. 2022).

The biodiversity of the Siwalik Mountains in Nepal remains inadequately studied (Chettry et al. 2021), which may partly explain why these newly discovered species of *Cyrtodactylus* have remained unnoticed until now. In recent years, only a limited number of herpetological surveys have been conducted in this region, yet they have resulted in notable findings, including the first national records of *Varanus salvator* (Laurenti, 1768) and *Takydromus sikkimensis* Günther, 1888 from the eastern Siwalik Mountains of Nepal (Bhattarai et al. 2020; Gautam et al. 2022).

Geographically, the Siwalik Mountains cover 12.8% of Nepal’s area (Gautam et al. 2022). Subedi et al. (2021) noted that approximately 55% of the habitats within Siwalik Mountains of Nepal are vulnerable to anthropogenic activities such as habitat destruction, encroachment, non-engineered road construction, and stochastic events such as forest fires, erratic rainfall, and landslides. Subedi et al. (2021) proposed eight biodiversity ‘pockets’, based on faunal richness in the Siwalik Mountains, and called for conservation actions to be prioritised in those areas. However, both *C. makwanpurgadhiensis* sp. nov. and *C. chure* sp. nov. are distributed outside these conservation priority pockets and protected area networks, in other ecologically sensitive areas. The designation of the type localities and their habitats of newly described species as other effective area-based conservation measures (OECMs) may contribute to long term biodiversity conservation. The OECMs are sites outside of the protected areas that deliver long term conservation of biodiversity and contribute to the implementation of the Kunming-Montreal Global Biodiversity Framework (Convention on Biological Diversity 2022; Jonas et al. 2024).

The discovery of the two new species of *Cyrtodactylus* from central Nepal reported in this study, after the recent description of three new *Cyrtodactylus* species by Bhattarai et al. (2025), demonstrates that surveys in new areas yield undescribed species. Detailed surveys including integrative taxonomy, combining morphological and genetic data, as well as more rigorous statistical analyses of morphology are needed to uncover their true diversity in Nepal, which would be crucial to inform and plan strategies for biodiversity conservation.

## Supplementary Material

XML Treatment for Cyrtodactylus makwanpurgadhiensis

XML Treatment for Cyrtodactylus chure

## ﻿Additional information

### Use of AI

No use of AI was reported.

### Author ORCIDs

Santosh Bhattarai https://orcid.org/0000-0001-7615-1954

Bivek Gautam https://orcid.org/0000-0001-5253-7801

Bishal Prasad Neupane https://orcid.org/0009-0000-3450-5195

Akshay Khandekar https://orcid.org/0000-0002-7956-089X

Tejas Thackeray https://orcid.org/0000-0002-9981-8763

Ishan Agarwal https://orcid.org/0000-0001-9734-5379

Ashley R. Olson https://orcid.org/0000-0002-3753-4482

Fiona Hogan https://orcid.org/0000-0001-6934-3720

Wendy Wright https://orcid.org/0000-0003-3388-1273

### Data availability

All of the data that support the findings of this study are available in the main text.

## ﻿Appendix 1

**Material examined.** Museum abbreviations are as follows: Bombay Natural History Society, Mumbai, India (BNHS); Centre for Ecological Sciences, Indian Institute of Science, Bangalore, India (CES); Natural History Museum Kathmandu, Nepal (NHM); Museum für Naturkunde, Leibniz-Institut für Evolutions- und Biodiversitätsforschung, Berlin, Germany (ZMB); Muséum d’Histoire naturelle, Geneva, Switzerland (MHNG); Zoologische Staatssammlung München, Munich, Germany (ZSM).

*Cyrtodactylus annapurnaensis*. Holotype, NHM 2023/367 (SB029), adult male; paratypes, NHM 2023/368 (SB030) and NHM 2023/369 (SB031), adult females, NHM 2023/370 (SB032) adult male, from Lwang; ZMB 57898, adult male, from Birethanti; ZMB 61691 (field no. 6670), adult male, from 1 km east of Naudanda; ZMB 61692–61694 (field nos. 6671–6673) adult females, from the eastern outskirts of Naudanda; all from Kaski District, Gandaki Province, Nepal.

*Cyrtodactylus bhupathyi*. Holotype, BNHS 2255, adult female; paratype BNHS 2256, adult female; from near Bagdogra, Darjeeling District, West Bengal, India.

*Cyrtodactylus chamba*. Holotype, BNHS 2332, adult male; paratypes, BNHS 2330, adult male, BNHS 2333, juvenile male, BNHS 2331, BNHS 2334, and BNHS 2335, adult females, from near Chamba in Chamba District, Himachal Pradesh, India.

*Cyrtodactylus chitwanensis*. Holotype, NHM 2023/376 (SB052), adult male; paratypes, NHM 2023/364 (SB026), NHM 2023/365 (SB027), and NHM 2023/366 (SB028), adult females, NHM 2023/377 (SB053), adult male, and NHM 2023/378 (SB054), subadult male; all from Bandipur, Tanahun District, Gandaki Province; NHM 2023/362 (SB024) and NHM 2023/363 (SB025), adult females, from Kabilas, Chitwan District, Bagmati Province, Nepal.

*Cyrtodactylus fasciolatus*. CES09/1269–1271 from near Tattapani; CES09/1337–1339, from near Subathu, both in Shimla District, Himachal Pradesh, India.

*Cyrtodactylus gubernatoris*. BNHS 2207, adult male, BNHS 2208–2210, adult females, from near Singtam, East Sikkim District, Sikkim, India. http://www.reptile-database.org

*Cyrtodactylus himalayicus*. Holotype, ZSIK 15716, adult male, from Kurseong; ZSIK 19546, adult female from Gopaldhara; both from Darjeeling District, West Bengal, India.

*Cyrtodactylus karanshahi*. Holotype, NHM 2023/372 (SB034), adult male; paratypes, NHM 2023/371 (SB033), NHM 2023/373 (SB035), and NHM 2023/375 (SB037), adult males, NHM 2023/374 (SB036), adult female; from on the way from Philim to Chisapani, Gorkha District, Manaslu Conservation Area Gandaki Province, Nepal.

*Cyrtodactylus lawderanus*. CES09/ 1253–1256, Nahan-Renuka road, Sirmaur District; CES09/ 1262 Sadhupul, Solan District; CES09/ 1264–1266, near Jutogh, Shimla District; CES09/ 1268 Shimla-Pujarli Road, Shimla District; CES09/ 1275–1281, Aut, Mandi District; CES09/ 1285–1288 Kangra-Jawala Mukhi Road, Kangra District; CES09/ 1288 Sujanpur-Tira, District; CES09/ 1335 Rewalsar, Mandi District; all from Himachal Pradesh. CES09/ 1330, near Mansar, Udhampur District, Jammu and Kashmir, India; CES09/ 1343–1344, Almora, Almora District, Uttarakhand, India.

*Cyrtodactylus martinstolli*. Holotype, MHNG 2590.09, adult male; paratypes, MHNG 2590.10–33, from the road between Ilam town and Pawakhola [=Puwakhola] village (1200 to 1300 m asl.), Ilam District; MHNG 2590.35, adult male and ZISP 20685, adult female (holotype and paratype of *C. markuscombaii* respectively), from the road between Ilam town and Puwakhola village (1200 to 1300 m asl.), Ilam District; ZSM 0587/2012 (ex SH 2306), same data as for the holotype of *C. martinstolli*; NHM 2023/356 (SB014), adult male, NHM 2023/357 (SB015), NHM 2023/358 (SB016), NHM 2023/359 (SB017), adult females, from Dobate, between Ilam market and Puwakhola, Ilam District, Koshi Province, Nepal.

*Cyrtodactylus nepalensis*. Holotype, ZSM 854/2012 (originally SHHS 1998/33, VW D 94/14 (Fuhlrott-Museum Wuppertal), VW-D 94114, adult male (transferred to ZSM in 2012), from “Sakaye…close to Dipayal” [=Sakayal Village]; additional material, NHM 2023/360 (SB038), adult male, and NHM 2023/361 (SB039), adult female, from Sakayal, Dadeldhura District, Sudurpaschim Province, Nepal.
